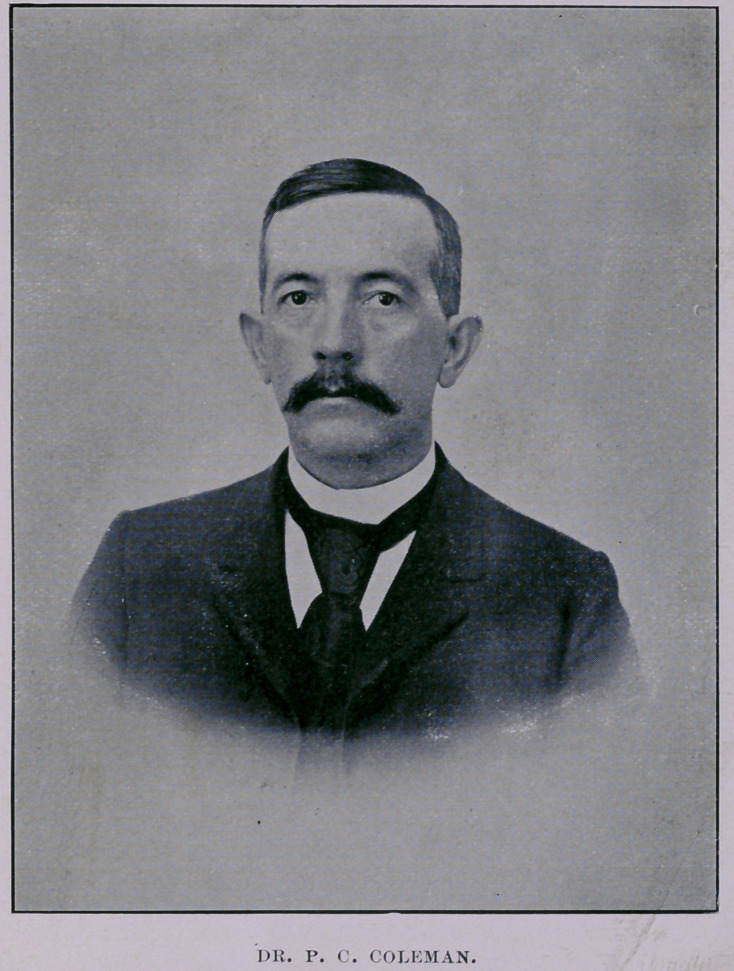# Biographical—Dr. P. C. Coleman

**Published:** 1895-05

**Authors:** 


### BIOGRAPHICAL.

### DR. P. C. COLEMAN, PRESIDENT-ELECT TEXAS STATE MEDICAL ASSOCIATION.

Dr. Preston Chiles Coleman, of Colorado county, Texas, whose portrait we have the pleasure of presenting to our readers herewith, is a Tennesseean by birth. He is a son of Dr. Waller Preston and Fannie Black-Preston, both of Scotch-Irish lineage. His early education was received in the common schools of Tennessee, and at the age of 18, he begun the study of medicine. He read at night and at such leisure times as his labors on the family farm afforded. After about two years of this course of reading, he went to Nashville and attended a course of medical lectures at the Medical Department, University of Nashville. That was the session of 1872–3. At the close of the session he resumed work upon the farm, acquiring there and then a strong physical development and vigorous health. Entering the Medical Department, University of Louisville, the next session, he took a second course. Was graduated M. D., in the spring of 1874.

Dr. Coleman is a physician by birth and heredity, we may say. He descended from a line of ancestry amongst whom were many doctors. His relatives were physicians, with few exceptions; uncles and cousins. His father was a classical scholar and a physician of fine attainments, and up to the date of his death in 1870, was the leading practitioner in the section of the State where he resided. Many of the relatives also attained distinction in the profession. Notably the Yandells, of Louisville, who are cousins of Dr. Coleman.

After receiving his diploma, Dr. Coleman located at his old home, and took up the work where his father had left it at his death. Here he practiced till 1883, when being seized with the "Texas fever," he removed to Colorado county—then away out west, on the frontier. In the meantime he had been bereaved of his young wife, to whom he was married, two years after graduating (in 1876). Her maiden name was Bettie Mitchel; she died in 1882.

Settled in his new home, and having determined to remain there, Dr. Coleman again married; his second wife being Miss Lucy Ham, of Tyler, Texas; they were married in April, 1885. There have been born to them five children; three girls and two boys.

DR. P. C. COLEMAN.

Believing in organization in medicine, the doctor set a praiseworthy example by immediately joining his county medical society upon entering upon the practice, and also joined the Tennessee State Medical Association; and during his residence in Tennessee he never missed a meeting when it was possible to be present. We may say the same of his connection with the Texas State Medical Association. Few members have manifested the same zeal and devotion to the interests of the State Medical Association, and none could have been more faithful and punctual in attendance. He joined the Texas State Medical Association in April, 1885, and has attended every *annual meeting*, having to travel several hundred miles each way in order to do so—say when the meeting was held in Houston or Galveston. This record, we dare say, is without a parallel.

Moreover, Dr. Coleman has shown an unswerving devotion to the Association's every interest, cheerfully working in every capacity to which he has been assigned. He has been Chairman of Section, has served—and well—on various committees, and for four years was on the judicial council at the stormiest period of the Association's history. He has, indeed, fairly won the honor bestowed upon him unanimously by an admiring and appreciative constituency, and he will most worthily wear it. He has also contributed valuable papers to several of the departments, all of which are presented in the Transactions of the Association; most of them have been reproduced in the TEXAS MEDICAL JOURNAL, the New York *Medical Record* and other leading journals.

In 1892 Dr. Coleman was elected First Vice-President of the Texas State Medical Association, and has served as delegate to the American Medical Association, of which body he is an active member.

Dr. Coleman will make a successful and popular president. He will work for the up building and permanency of the Association, and is already, by correspondence and personally, endeavor ing to enlist a large number of the influential men who have for various reasons, heretofore held aloof from the organization.

Dr. Coleman is a graceful and forceful speaker, and will make us a president of whom we will have occasion to be proud.

## Figures and Tables

**Figure f1:**