# Errata

**Published:** 1895-06

**Authors:** 


**Errata.**--The JOURNAL regrets exceedingly the occurrence of several errors in the biography of Dr. Coleman, in our last issue. They were not discovered until too late to be corrected. It was stated that Dr. Coleman was the son of Dr. Walter Preston and Fannie Preston, the mistake being caused, no doubt, by the fact that Coleman's middle name is Preston. His father was Dr. Walter Coleman, a leading physician in Tennessee. We also located the Doctor in Colorado county, instead of Colorado City, Mitchell county.

